# Medium cut-off membrane expanded hemodialysis for Lithium removal: a case report

**DOI:** 10.3389/ftox.2025.1677299

**Published:** 2025-11-14

**Authors:** Katia M. Pérez del Valle, María Moran Magro, Daniel Gaitán Tocora, Nerea Begoña Boldoba, Carmen Benito Puncel, Alberto Silva Obregón, José R. Rodríguez Palomares, Gabriel de Arriba de la Fuente

**Affiliations:** 1 Nephrology Department, Hospital Universitario de Guadalajara, Guadalajara, Spain; 2 Intensive Medicine Department, Hospital Universitario de Guadalajara, Guadalajara, Spain; 3 Department of Medicine and Medical Specialties, University of Alcalá (UAH), Madrid, Spain

**Keywords:** Lithium, expanded hemodialysis, HDX, medium cut-off membrane, toxicity, extracorporeal blood purification

## Abstract

**Background:**

Lithium remains a first-line treatment for bipolar disorder, though its narrow therapeutic window poses a significant risk of toxicity. Severe intoxication can lead to neurologic, renal, gastrointestinal, and endocrine complications. Extracorporeal therapies are the mainstay in managing life-threatening cases, particularly when serum concentration exceed 4.0 mmol/L or when renal function is impaired. Intermittent hemodialysis (HD) is the recommended extracorporeal treatment due to lithium’s low molecular weight and minimal protein binding. Expanded hemodialysis (HDx) with medium cut-off (MCO) membranes, designed to enhance solute clearance, may represent a promising alternative.

**Case Report:**

We present a case of acute lithium intoxication in a 48-year-old male with a history of recurrent suicidal behavior and chronic lithium therapy. The patient arrived at the emergency department in a deep coma (GCS 3), with a serum lithium concentration of 4.5 mmol/L and preserved renal function. He underwent two intermittent HDx sessions using a Theranova® TH-400 MCO membrane. Serum lithium concentration declined to 1.6 mmol/L after the first session and progressively to 0.1 mmol/L within 72 h. No treatment-related complications were observed.

**Conclusion:**

This case described the potential utility of HDx with the MCO membrane (Theranova® 400) in managing severe lithium poisoning, achieving effective and sustained drug clearance. While HDx-MCO may offer a viable alternative to conventional HD, especially in settings where enhanced solute removal is desirable, further studies are needed to determine its efficacy and clinical role in lithium toxicity management.

## 1 Introduction

Lithium continues to be regarded as the first-line pharmacological agent for the long-term management of recurrent bipolar disorder (Vieta and Sanchez-Moreno, 2008). As a monovalent cation analogous to sodium, lithium’s precise mechanism of action remains incompletely understood. It was initially approved by the U.S. Food and Drug Administration (FDA) in the 1970s for its mood-stabilizing properties, specifically in the treatment of acute manic episodes (Gajwani et al., 2006). Despite its efficacy as a potent antimanic agent, lithium is characterized by a narrow therapeutic window, necessitating careful monitoring to avoid toxicity (Ware et al., 2016).

Despite its well-established clinical efficacy, lithium’s therapeutic benefits must be carefully weighed against its substantial adverse effect profile and notably narrow therapeutic range (Hedya et al., 2025).

Lithium intoxication presents with neurologic symptoms such as coarse tremor, hyperreflexia, nystagmus, ataxia, and altered mental status, which may rarely persist beyond 12 months (Ott et al., 2016). Chronic use is associated with nephrotoxicity, including nephrogenic diabetes insipidus, sodium-wasting nephropathy, and nephrotic syndrome (Davis et al., 2018). Cardiovascular effects are generally mild, with T wave flattening and sinus node dysfunction being the most common conduction abnormalities, all typically reversible (Canan et al., 2008). Gastrointestinal symptoms occur early, especially in acute overdose (Mohandas and Rajmohan, 2007). Endocrine disturbances include hypothyroidism due to inhibited thyroid hormone synthesis; hyperthyroidism is less common but may exacerbate toxicity (Kibirige et al., 2013).

However, accurate assessment of lithium toxicity requires consideration of several factors, including the ingested dose, time since ingestion, presence of co-ingestants, and whether the exposure was intentional or accidental. Notably, clinical manifestations of lithium toxicity frequently do not correlate directly with serum lithium concentrations (Foulser et al.).

Lithium poisoning is classified into three types: acute (in lithium-naïve individuals), acute-on-chronic (in patients on maintenance therapy with acute overdose), and chronic (due to dose escalation, renal impairment, or interactions reducing lithium clearance) (Decker et al., 2015).

Management of severe lithium toxicity begins with discontinuation of lithium and supportive care, including intravenous isotonic saline for volume expansion (Amdisen, 1988; Khasraw et al., 2012). Activated charcoal is generally ineffective, as lithium does not bind to it, though it may be used when co-ingestants are suspected (Favin et al., 1988). Gastric lavage may be considered in early-presenting cases with immediate-release formulations, while whole-bowel irrigation is recommended for sustained-release or massive ingestions (Haussmann et al., 2015). However, no decontamination strategy has been definitively shown to improve outcomes (Bretaudeau et al., 2013).

Sodium polystyrene sulfonate has been suggested to aid lithium elimination but lacks demonstrated clinical efficacy (MacLeod-Glover and Chuang, 2020).

Intermittent hemodialysis is the most effective method of lithium removal, particularly in cases of severe toxicity or renal impairment, due to lithium’s low molecular weight and limited protein binding (Amdisen, 1988; Waring, 2006).

The Extracorporeal Treatments in Poisoning (EXTRIP) Workgroup is an international consortium of experts dedicated to formulating evidence-based guidelines for the application of extracorporeal therapies (ECTRs) in the management of toxicological emergencies (Ghannoum et al., 2011; Lavergne et al., 2012).

Based on a systematic review and expert consensus, EXTRIP Workgroup recommended hemodialysis (HD) as the treatment of choice in severe cases of lithium poisoning, despite the overall low quality of available evidence (Decker et al., 2015).

Given lithium’s favorable physicochemical properties, it may be hypothesized that expanded hemodialysis (HDx) with medium cut-off (MCO) membranes may be an effective modality for lithium removal in cases of poisoning.

We present a rare case of acute, severe lithium intoxication requiring HDx with an MCO at Hospital Universitario de Guadalajara (Guadalajara, Spain).

## 2 Case report

A 48-year-old male with a past psychiatric history of mixed personality disorder and recurrent major depressive disorder was admitted to the emergency department (ED) in a state of deep coma (GCS 3), unresponsive to flumazenil and naloxone. He had a long-standing history of suicidal behavior requiring multiple prior psychiatric hospitalizations. Home medications included lithium 400 mg every 8 h, quetiapine, sertraline, and benzodiazepines. The patient was an active smoker with a documented history of substance use (cocaine, alcohol, and cannabis) and lacked stable family support. His baseline frailty score was 3–4. At admission, serum lithium concentration was 4.5 mmol/L, indicating severe toxicity (>2.5 mmol/L), though renal function was preserved (eGFR >120 mL/min, creatinine 0.66–0.72 mg/dL). Laboratory findings showed normal serum osmolality (288 mOsm/kg), mild hyponatremia, mild hypophosphatemia, and normal magnesium concentration. Additional findings included elevated C-reactive protein (6.8 U/L), mild normocytic anemia, and transient neutrophilic leukocytosis.

Given the patient’s Glasgow Coma Scale score of three and history of drug use, an initial differential diagnosis was conducted to identify potential metabolic, toxic, or structural causes. Capillary blood glucose, thyroid function (TSH), urine toxicology screening, and serum lithium concentration was promptly obtained. Urine screening was negative for other substances, TSH values were within normal limits, and cranial CT imaging revealed no structural abnormalities. The markedly elevated lithium concentration (4.5 mmol/L) confirmed acute-on-chronic lithium intoxication as the primary etiology of the neurological impairment.

The patient’s pertinent demographic, clinical, and biochemical findings at intensive care unit (ICU) admission are summarized in Table 1.

**TABLE 1:** Summary of key demographic, clinical, and biochemical parameters of the patient at Intensive Care Unit (ICU) admission.

Demographic characteristics	
Sex	Male
Age	48 years
Race	Caucasian

Physical Examination	
Conscious and oriented in all three spheres Eupneic, good general condition No skin lesions Pupils: Medium, normoreactive No lymphadenopathy, no goiter, no trauma signs Cardiac auscultation: Rhythmic, no murmurs Pulmonary auscultation: No added sounds Abdomen: Soft, depressible, non-tender, no peritoneal irritation signs ○ Murphy: Negative, Blumberg: Negative ○ No masses or organomegaly ○ Bowel sounds present, no bruits Lower limbs: No edema, no signs of DVT or venous insufficiency Pulses: Symmetrical and present	

Clinical Characteristics Before HDx-MCO	
Reason for Admission	Drug-induced coma Severe lithium poisoning
Litihum, mmol/L	4.5^b^
Vital signs SBP, mmHg DBP, mmHg HR, bpm Temperature RF, breaths pm	130 80 110 35.6 Celsius degrees 20
Arterial blood gas analysis FiO_2_ pH PO_2_, mmHg PCO_2_, mmHg HCO_3_, mmol/L Lactate, mmol/L Ionized Calcium, mmol/L	100%^a^ 7.28 115 53 24.9 0.5 1.27
Blood analysis Hemoglobin, g/dL Hematocrit, % MCV, fL Leukocytes, mm^3^ Platelets, mm^3^ RDW, % INR PT, seconds PTT, seconds Fibrinogen, mg/dL	12.3 37.2 91.9 9,900 227,000 14.3 1.15 12.82 31 266

Biochemical Characteristics Before HDx-MCO	
Biochemistry Glucose, mg/dL Creatinine, mg/dL Urea, mg/dL Na, mmol/L K, mmol/L Cl, mmol/L P, mmol/L Mg, mmol/L Ca, mmol/L CRP, U/L Procalcitonin, ng/mL Blood osmolarity, mOsm/kg	100 0.72 17 136 3.7 111 3.6 2.1 9.3 6.8 0.04 293
Liver profile AST, U/L ALT, U/L GGT, U/L LDH, U/L ALP, U/L Total bilirubin, mg/dL Albumin, g/L Total proteins, g/L	15 10 17 123 42 0.4 45 49.5

^a^ In mechanical ventilation.

^b^ Therapeutic range: 0.8 – 1.2 mmol/L

HDx, Expanded hemodialysis; MCO, Medium cut-off membrane; Bpm, Beats per minute; FiO_2_, Fraction of Inspired Oxygen; IVM, Invasive mechanical ventilation; MCV, Mean Corpuscular Volume; RDW: Red Cell Distribution Width; INR: International Normalized Ratio; PT, Prothrombin Time; PTT, Activated Partial Thromboplastin Time; RCP, Reactive C Protein; AST, Aspartate aminotransferase; ALT, Alanine transaminase; GGT, Gamma-glutamyl transferase; LDH, Lactate dehydrogenase; ALP, Alkaline phosphatase.

The patient underwent two intermittent HDx-MCO membrane sessions, administered 12 h apart. Treatment parameters included a blood flow rate of 250 mL/min, an ultrafiltration rate of 200 mL/h, and systemic anticoagulation with heparin at a dose of 1,000 IU. The dialysate composition was specified as 211–25 with potassium supplementation. Dialysis was performed using the Theranova TH-400 dialyzer (Baxter Healthcare Corporation, Deerfield, IL, USA), a MCO membrane designed to enhance clearance of middle and large molecular weight solutes (Mitchell et al., 2023; Kawanishi, 2024).

**FIGURE 1:**
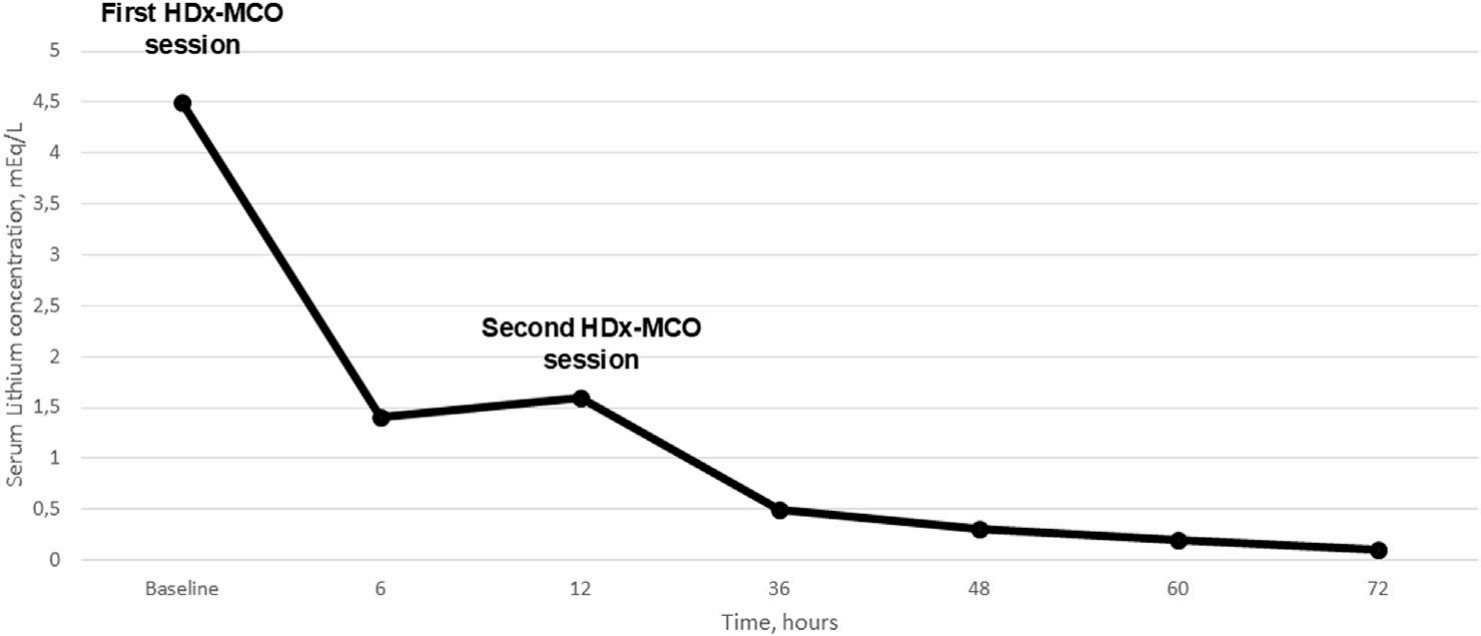
Serial measurements of serum lithium were conducted throughout the patient’s clinical management. The therapeutic protocol included two sessions of expanded hemodialysis utilizing a medium cut‐off membrane (HDx‐MCO), administered at a 12‐hour interval. The initial serum lithium concentration was markedly elevated at 4.5 mEq/L. Following the first HDx‐MCO session, the level declined to 1.4 mEq/L at 6 hours and 1.6 mEq/L at the 12‐hours after the first HDx‐MCO session. A continued reduction was observed after the second session, with concentrations decreasing sequentially to 0.5 mEq/L at 36 hours, 0.3 mEq/L at 48 hours, 0.2 mEq/L at 60 hours, and ultimately reaching 0.1 mEq/L at 72 hours post-initiation of extracorporeal therapy. This profile reflects the effective clearance of lithium achieved through the HDx-MCO modality.

Following the first HDx-MCO session, serum lithium concentration decreased from 4.5 mmol/L to 1.6 mmol/L at 12 h. After the second session, concentration declined progressively to 0.5 mmol/L at 36 h, 0.3 mmol/L at 48 h, 0.2 mmol/L at 60 h, and 0.1 mmol/L at 72 h. These findings indicated effective and sustained lithium clearance with sequential HDx-MCO treatments (Figure 1). During the ICU stay, the patient received empirical antibiotic therapy with amoxicillin/clavulanate for suspected bronchoaspiration, although chest radiography revealed no evidence of pneumonic infiltrates. This clinical context, together with the systemic inflammatory response inherent to critical illness, likely contributed to the observed progressive increase in CRP levels. A mild decline in hemoglobin was noted and attributed to hemodilution secondary to fluid resuscitation and repeated blood sampling, with no signs of active bleeding. These changes were not considered related to the HDx sessions, as no significant blood losses associated with the dialysis technique were observed.

Clinically, the patient exhibited progressive neurological improvement and was successfully extubated 48 h later, achieving full recovery of consciousness without residual neurological deficits at ICU discharge. During hospitalization, no neurological or cardiovascular complications were observed. Lithium therapy was permanently discontinued, and psychiatric management was transitioned to alternative mood-stabilizing medications. Outpatient follow-up with psychiatry and nephrology confirmed the absence of long-term sequelae related to either the intoxication or the treatment modality.

Table 2 details the progression of critical biochemical markers monitored throughout the patient’s clinical course.

**TABLE 2:** Overview of different biochemical parameters throughout the study follow-up.

Parameter	Baseline	Hour-6	Hour-12	Hour 36	Hour-48	Hour-72
Leukocytes, mm^3^	10,000	9,900	11,400	13,700	10,600	5,700
Hemoglobin, g/dL	13.1	12.3	12.1	12.1	11.5	11.5
Platelets, mm^3^	224,000	227,000	221,000	249,000	181,000	184,000
Neutrophils, mm^3^	7,800	7,900	9,400	11,600	8,700	3,500
Glucose, mg/dL	100	134	122	145	147	121
Creatinine, mg/dL	0.72	0.49	0.61	0.68	0.64	0.64
Urea, mg/dL	17	<6	7	13	11	12
Na, mmol/L	136	138	138	149	144	142
K, mmol/L	3.7	3.3	3.4	4.0	3.9	3.3
Cl, mmol/L	111	110	112	121	117	112
P, mmol/L	3.6	1.5	2.6	2.7	2.4	2.3
Mg, mmol/L	2.1	1.8	1.7	2.1	1.9	1.9
Ca, mmol/L	9.3	-	8.2	-	-	-
CRP, mg/L	7.3	6.8	26.7	170.7	168.3	127.8
Blood osmolarity, mOsm/kg	293	-	-		-	293
AST, U/L	16	-	16	14	14	10
ALT, U/L	11	-	10	10	9	8
GGT, U/L	18	-	-	17	19	23
LDH, U/L	160	-	121	183	201	125
ALP, U/L	42	-	-	62	78	60
Total bilirubin, mg/dL	0.4	-	0.6	0.4	0.4	0.5
Albumin, g/L	45	-	29.5	32.6	30.5	32
Total proteins, g/L	49.5	-	45.9	52.1	50.7	54.1

CRP, Reactive C Protein; AST, aspartate aminotransferase; ALT, alanine transaminase; GGT, Gamma-glutamyl transferase; LDH, lactate dehydrogenase; ALP, alkaline phosphatase.

## 3 Discussion

Lithium poisoning is classified into acute, chronic, and acute-on-chronic forms. Acute toxicity typically follows a single large ingestion, with predominant gastrointestinal manifestations due to lithium’s enteral absorption (Decker et al., 2015). Chronic toxicity arises more commonly and is often related to impaired renal elimination, secondary to factors such as volume depletion, infections, or pharmacologic interactions. The acute-on-chronic presentation involves overlapping clinical features of both acute and chronic exposures (Decker et al., 2015).

Acute-on-chronic lithium poisoning is associated with a less favorable prognosis compared to cases without prior exposure, primarily due to a higher incidence of central nervous system involvement (Hedya et al., 2025; Ott et al., 2016). In this context, approximately 5% of patients require orotracheal intubation, and seizures occur in about 3% (Hedya et al., 2025; Ott et al., 2016). Neurotoxicity appears to be at least partially dose-dependent, with more severe manifestations observed at higher serum lithium concentration (Hedya et al., 2025; Ott et al., 2016). Accordingly, the 48-year-old male presented in this case, with a lithium concentration of 4.5 mmol/L in the setting of acute-on-chronic toxicity, could be considered at elevated risk for severe neurologic complications.

The management of lithium toxicity involves supportive care, specifically protecting the airway if the patient has altered mental status, discontinuation of lithium exposure and enhancement of renal elimination (Hedya et al., 2025; Ott et al., 2016). The EXTRIP workgroup published guidelines in 2015 on the use of extracorporeal therapies for lithium poisoning, based on a review of 166 studies involving 418 patients (Decker et al., 2015). They recommended extracorporeal treatment in severe cases, particularly with impaired renal function, lithium concentration >4.0 mmol/L, or the presence of altered consciousness, seizures, or life-threatening arrhythmias. Treatment is also suggested for lithium concentration >5.0 mmol/L (whatever the kidney function), marked confusion, or if lithium clearance is expected to exceed 36 h. Therapy should continue until clinical improvement or serum lithium falls below 1.0 mmol/L (Decker et al., 2015).

Lithium’s low molecular weight, small volume of distribution, and minimal protein binding enable efficient plasma clearance by hemodialysis (Decker et al., 2015; King et al., 2019). However, unlike most toxins, lithium redistributes from intracellular compartments to plasma through sodium channels, resulting in slower equilibration compared to substances that diffuse freely across membranes. Consequently, multiple hemodialysis sessions are often required due to plasma lithium rebound occurring hours post-treatment (Decker et al., 2015; King et al., 2019). In this case, serum lithium was measured before and after each HDx-MCO session, with no rebound observed.

In this case, an HDx-MCO membrane (Theranova®; Baxter International Inc., Deerfield, IL, USA) was employed for lithium removal. Given lithium’s low molecular weight, limited volume of distribution, and minimal protein binding, it is well-suited for clearance via HDx-MCO (Mitchell et al., 2023; Kawanishi, 2024). Compared to conventional high-flux hemodialysis and hemodiafiltration, HDx-MCO offers enhanced elimination of larger middle molecules while reducing albumin loss (Mitchell et al., 2023; Kawanishi, 2024). A recent meta-analysis confirmed superior clearance of κ and λ free light chains with HDx-MCO and emphasized its improved albumin preservation relative to hemodiafiltration (Zhao et al., 2022).

Albumin loss in our study was negligible and aligned with data from earlier clinical studies (Kirsch et al., 2017; Maduell et al., 2022; Hagemann et al., 2023). The membrane’s design enabled a reduction in extracorporeal treatment duration, thereby limiting ionic and metabolic disturbances, regardless of vascular access flow, and offering straightforward clinical application (Kirsch et al., 2017; Maduell et al., 2022; Hagemann et al., 2023). Furthermore, by enhancing the clearance of a broader range of toxins, HDx with MCO membranes may mitigate some adverse outcomes of conventional hemodialysis, including dialytrauma (Hagemann et al., 2023; García-Prieto et al., 2023).

To our knowledge, this is the first report detailing the application of HDx-MCO in lithium poisoning. Although clinical improvement was noted post-treatment, the lack of controlled trials limits definitive conclusions. As highlighted by Decker et al. (Decker et al., 2015), robust randomized data on extracorporeal interventions for lithium toxicity remain scarce. In this case, the use of HDx-MCO was considered based on its superior clearance of inflammatory mediators and middle molecules compared with conventional high-flux dialyzers (Zickler et al., 2017; Kandi et al., 2022). We hypothesized that, by reducing interstitial inflammation and optimizing fluid dynamics, HDx-MCO might theoretically attenuate concentration gradients between compartments and mitigate the risk of delayed lithium redistribution (rebound) (Decker et al., 2015). Nevertheless, this hypothesis cannot be demonstrated by a single case observation, and no clinical studies to date have directly assessed the impact of HDx-MCO on lithium rebound. Thus, this potential advantage should be interpreted as a plausible but unproven physiological rationale rather than a confirmed clinical effect.

In conclusion, lithium’s narrow therapeutic window has remained a management challenge, with toxicity representing a persistent clinical concern. Extracorporeal therapies have played a key role in cases of severe poisoning by reducing the duration of central nervous system exposure to toxic concentrations. Their use has been particularly warranted in the presence of renal impairment, neurologic symptoms, or serum lithium concentration exceeding 4.0 mmol/L. In this case, HDx-MCO proved effective in achieving a rapid reduction in serum lithium concentration. Further research is needed to clarify its efficacy and potential role in the management of lithium toxicity.

## Data Availability

The raw data supporting the conclusions of this article will be made available by the authors, without undue reservation.
